# Apathy in Parkinson's Disease: Distinguishing Overlapping Symptoms Via Network Analysis

**DOI:** 10.1002/mdc3.70698

**Published:** 2026-06-02

**Authors:** Joseph Seemiller, Abhimanyu Mahajan, Christopher B. Morrow, Joseph F. McGuire, Dawn Bowers, Kelly A. Mills, Gregory M. Pontone

**Affiliations:** ^1^ Department of Neurology Pennsylvania State University‐Milton S. Hershey Medical Center Hershey Pennsylvania USA; ^2^ Gardner Family Center for Parkinson's Disease and Movement Disorders, University of Cincinnati Cincinnati Ohio USA; ^3^ Department of Psychiatry and Behavioral Sciences Johns Hopkins School of Medicine Baltimore Maryland USA; ^4^ Department of Clinical and Health Psychology and Neurology Fixel Institute for Neurological Diseases, University of Florida Gainesville Florida USA; ^5^ Department of Neurology Johns Hopkins School of Medicine Baltimore Maryland USA; ^6^ Department of Neurology University of Florida College of Medicine Gainesville Florida USA

**Keywords:** Parkinson's disease, apathy, fatigue, sleepiness

## Abstract

**Background:**

Anxiety, fatigue, and excessive daytime sleepiness (EDS) frequently co‐occur in Parkinson's disease (PD) and can influence the clinical determination of apathy.

**Objective:**

To distinguish patient‐reported apathy from other non‐motor symptoms.

**Methods:**

Individuals with PD meeting UK Brain Bank criteria underwent paired OFF‐ and ON‐ dopaminergic medication assessments, including the Movement Disorder Society‐Unified Parkinson's Disease Rating Scale (MDS‐UPDRS), Hamilton Anxiety and Depression Rating Scales (HAM‐A, HAM‐D), Symbol Digit Modalities Test, Stroop, and Scales for Outcomes in Parkinson's Disease‐Autonomic Dysfunction assessment. Medication‐related changes in anxiety, depression, and cognition were defined using established minimally clinically important differences. Apathy, fatigue, and sleepiness were determined based on MDS‐UPDRS Part I scores ≥1. Associations were examined using chi‐squared tests, multivariable ordinal logistic regression, and symptom‐network analyses.

**Results:**

Among 199 participants (36–85 years, 61% male), 90% (53/59) of those with apathy also reported both fatigue and sleepiness. Exploratory network analysis grouped apathy with anxiety and depression, whereas fatigue clustered with somatic symptoms (eg, pain, constipation) and daytime sleepiness remained peripheral. Apathy was independently associated with medication‐related change in anxiety after adjustment for fatigue and sleepiness (*P* < 0.001), while fatigue was associated with autonomic dysfunction (*P* < 0.05).

**Conclusion:**

Despite overlapping presentation, apathy in PD closely tracks affective change linked to dopaminergic state, whereas fatigue and sleepiness reflect broader somatic burden. These distinctions may support more specific recognition and phenotyping of apathy.

Apathy is a clinical syndrome manifesting as reduced motivation, interest, and goal‐directed behavior. Apathy is seen in multiple neurodegenerative disorders,^1^, ^2^, ^3^ affects up to 40% in Parkinson's disease (PD)^4^ and is associated with lower quality of life^5^ and increased mortality.^6^ Yet, in routine clinical practice, apathy frequently overlaps phenotypically with fatigue, excessive daytime sleepiness (EDS), mood symptoms, and cognitive complaints, obscuring its recognition.^5^, ^7^, ^8^, ^9^, ^10^, ^11^, ^12^, ^13^, ^14^ Distinguishing symptoms that mimic apathy and symptoms that are dopamine‐responsive is a critical step for both clinical care and research targeting specific mechanisms of apathy.

Apathy in PD reflects multifactorial neurotransmitter dysfunction. Dopamine state changes are associated with blunted reward sensitivity,^15^ and apathy in PD is higher off‐medication.^15^, ^16^ This is consistent with dopamine's role in reward processing and effort‐cost computation via mesocorticolimbic pathways.^17^, ^18^, ^19^ Psychomotor slowing, fatigue, and reduced initiation blur diagnostic boundaries with apathy, depression, and anxiety,^20^, ^21^ Yet, each can present in isolation, reflecting distinct underlying mechanisms.^22^ Anxiety is common with apathy in early, de novo PD, and pure apathy is frequently misdiagnosed as anxiety.^23^ Serotonergic contributions are illustrated by SSRI‐induced apathy, in which enhanced serotonergic tone can paradoxically attenuate motivational drive.^24^ Several mechanistic contributions to apathy have been proposed.

1. Cognitive mechanisms may reflect overlapping network and neurotransmitter dysfunction but may also contribute directly to apathy symptomatology. Executive dysfunction can increase the cognitive effort required to initiate behavior and present as apathy.^25^ This executive/motivational interface may explain reports of benefit with cholinergic augmentation.^26^
2. Fatigue and EDS are prevalent in PD (40–58%^5^, ^7^ and ~ 35%^9^ respectively), sharing a hypoarousal phenotype that frequently co‐occurs with apathy.^5^ Although EDS and fatigue likely diverge mechanistically,^27^ patients often conflate these complaints clinically.^28^ Beyond self‐report overlap, EDS and apathy may share a dopaminergic substrate: daytime sleepiness has been associated with nigrostriatal degeneration in early PD independent of motor severity and depression,^29^ while dopamine centrally regulates motivated behavior via frontostriatal reward circuitry implicated in apathy.^30^ The increased frequency of sleep disorders observed in patients with apathy further suggests phenotypic convergence.^14^
3. Dysautonomia is prevalent in PD and is associated with fatigue and daytime sleepiness,^31^, ^32^, ^33^ potentially contributing to fluctuations through shared arousal and interoceptive mechanisms.^34^, ^35^
4. Mesolimbic dopaminergic dysfunction in the anterior cingulate cortex and ventral striatum has been linked with apathy,^25^ shifting choices toward low‐effort, low‐reward options independent of motor impairment.^36^, ^37^ Neuroimaging evidence also implicates noradrenergic, glutamatergic, and serotonergic contributions to fatigue and hypoarousal, suggesting that multiple systems converge on overlapping fronto‐striatal and limbic networks.^38^, ^39^ Dopamine‐sensitive anxiety in PD is associated with altered fronto‐limbic‐striatal connectivity within these circuits and with reduced dopamine transporter availability, and fluctuations with medication‐state.^38^, ^39^

The primary goal of this study was to delineate how apathy, fatigue, and EDS diverge in their network‐structural organization in PD. Including fatigue and EDS provides a necessary physiological contrast to test whether arousal‐driven somatic symptoms occupy distinct network communities from affective and motivational deficits more specific to apathy. We hypothesized that apathy would cluster with its affective, behavioral, and cognitive domains, whereas fatigue and EDS would cluster with somatic and autonomic symptoms. We also examined whether dopamine medication‐related changes and dysautonomia differentially related to these symptom clusters. We hypothesized that apathy would specifically track with dopamine‐sensitive anxiety changes, consistent with shared cortico‐limbic circuitry.

## Methods

### Participants

The dataset was previously described.^39^, ^40^ In short, 200 participants were recruited with a diagnosis of idiopathic PD based on UK PD society brain bank criteria;^41^ participants were 65 years old on average (SD 7.71; range 36 to 85 years), mostly (61.0%) men, and 92.5% white. To avoid recruiting participants with Lewy body dementia, participants had more than 1 year of symptoms, and cases with a clinical diagnosis of dementia were excluded. This study represents a secondary exploratory analysis of this existing dataset.^39^

### Procedures for Data Collection

Participants completed assessments in the “off” state (≥12 h medication withdrawal) and again in their best “on” state following their usual morning medication dose. Ethical approval for this study was granted by The Johns Hopkins University Institutional Review Board. All participants provided written informed consent prior to participation. The study was conducted in accordance with the ethical standards of the 1964 Declaration of Helsinki. Cases of missing data are detailed in Appendix S1. Years of disease duration (YoD) were calculated from the date of initial diagnosis. Total levodopa equivalent daily dose (LEDD) was calculated for each participant.^42^

### Assessments

Measures performed in both off and on medication states were Movement Disorder Society‐Unified Parkinson's Disease Rating Scale (MDS‐UPDRS) Part III, Hamilton Anxiety Rating Scale (HAM‐A), Hamilton Depression Rating Scale (HAM‐D), Symbol Digit Modalities Test (SDMT), and Stroop. The following measures were performed only in the off‐medication state: MDS‐UPDRS Part I, which assessed the last 1 week of symptoms and Scales for Outcomes in Parkinson's Disease‐Autonomic Dysfunction (SCOPA‐AUT), which assessed the last 1 month of symptoms. Total testing spanned 3 hours, including the time to reach best ON (mean [SD] 72.28 [22.72] minutes).

Cognitive testing included oral versions of the SDMT^43^ and Stroop^44^ tests, as previously described.^39^, ^40^ Subjects were excluded from analysis if they did not tolerate repeat cognitive testing or if entering the study prior to the test's addition to the protocol (Appendix S1 and S2).

Mood was assessed using the HAM‐A^45^ and the HAM‐D.^46^ Both were modified to be answered for the current state (on or off dopamine medication).

Participants completed the full MDS‐UPDRS;^47^ the motor examination (Part III) was repeated before and after medication to capture OFF and ON states. Apathy, daytime sleepiness, and fatigue were considered present if Part I items 1.5, 1.8, and 1.13, respectively, were endorsed at least as “slight” (score ≥1). Part I was administered only in the Off‐medication state and, consistent with recommended administration instructions, with responses reflecting symptoms over the preceding week.

Dysautonomia was assessed using the SCOPA‐AUT, a well‐validated scale for measuring symptoms of autonomic dysfunction in PD over the past 1 month.^48^ The established cutoff score of 13.1 was used to define the presence of dysautonomia.^49^

Clinically meaningful change in cognitive or psychiatric measures in relation to dopamine medication was defined based on established minimally clinically significant important differences (MCID): change of 4 on the SDMT,^50^ 5.5 on Stroop color‐word interference naming,^51^ and 4 for HAM‐D.^52^ Given no widely accepted MCID for HAM‐A, we used 7.5 as described previously which is 0.5 standard deviations (SD) beyond the mean change in our population.^34^ Dopamine medication‐related change denotes the within‐visit difference between off‐ and on‐medication states.

### Network Analysis

Network modeling was used to explore conditional dependencies among non‐motor symptoms in a hypothesis‐generating approach. For network analysis, two participants were removed: one due to missing sleep‐related questions of the MDS‐UPDRS and one due to not completing the Hamilton depression rating scale. To improve network stability, several adjustments were made to the variables. Two items, MDS‐UPDRS 1.2 (psychosis) and 1.6 (dopamine dysregulation syndrome), were excluded as no participants received the two most severe scores. Additionally, the scoring range for all remaining MDS‐UPDRS Part I items was condensed from 0–4 to 0–3 by collapsing the two most severe categories to reduce sparse or empty cells in the ordinal responses and enhance stability of the EBICglasso network estimation.

The network was estimated using Spearman rank‐order correlation matrix using the bootnet package.^53^ A gaussian graphical model (GGM) was estimated using graphical LASSO (least absolute shrinkage and selection operator) with extended Bayesian information criterion (EBICglasso) for model selection, which creates a sparse and more interpretable network by shrinking weak edges to zero. The EBIC hypertuning parameter was set to 0.25 to favor sensitivity over specificity.^54^ For network graph visualization, a spring‐based network algorithm was used, with minimum line threshold 0.05 to reduce visual clutter. We assessed the network's stability by performing a case‐dropping bootstrap procedure with 5000 iterations to calculate the correlation stability (CS) coefficient. Node centrality was calculated to identify the most influential symptoms in the network. To identify distinct clusters of symptoms within the network, we performed community detection using the Leiden algorithm.^55^

### Statistical Analysis

We leveraged paired off/on‐medication assessments in mood and cognitive variables to dissect how apathy, fatigue, and daytime sleepiness differ.

All statistical analyses were conducted in R (v4.4.0; R Core Team 2021). A two‐tailed *P*‐value <0.05 was considered statistically significant for all analyses. While our regression models are exploratory, we also applied Benjamini‐Hochberg False Discovery Rate (FDR) correction for multiple comparisons.

First, chi‐squared tests evaluated the association between the presence of apathy and medication‐related changes in psychiatric (HAM‐D, HAM‐A) or cognitive (SDMT, Stroop) scores.

Second, three separate ordinal regression models examined predictors of apathy, fatigue, and daytime sleepiness using ordinal item scores of 0–4. Each model included covariates of age, sex, disease duration, and off‐medication MDS‐UPDRS Part III score, which were selected based on the hypothesized influence of demographic factors, disease duration, and motor severity on the three non‐motor outcomes. The primary predictors in each model included binarized indicators of clinically significant medication‐induced change in anxiety, depression, or cognition, as well as the presence of dysautonomia.

Given the strong adherence between fatigue and apathy in the network model, further sensitivity analyses using multiple linear regression assessed if the binarized presence of fatigue and daytime sleepiness were independently associated with apathy severity (0–4) after adjusting for the primary predictors. As part of these analyses, multicollinearity among predictors was assessed by calculating the variance inflation factor (VIF).

Another sensitivity analysis accounted for the potential medication‐related effects on symptoms: We added total LEDD and LEDD due to dopamine agonist medications as covariates in our regression models, given that their differential dopamine receptor affinities may have different effects on the mesolimbic component of apathy.

Finally, our primary definition of apathy included “slight” severity symptoms (score ≥1). To improve construct validity, we conducted additional sensitivity analyses assessing if our primary predictors were associated with clinically relevant apathy (item 1.5 score of ≥2).

## Results

### Symptom Co‐Occurrence

**Figure 1:**
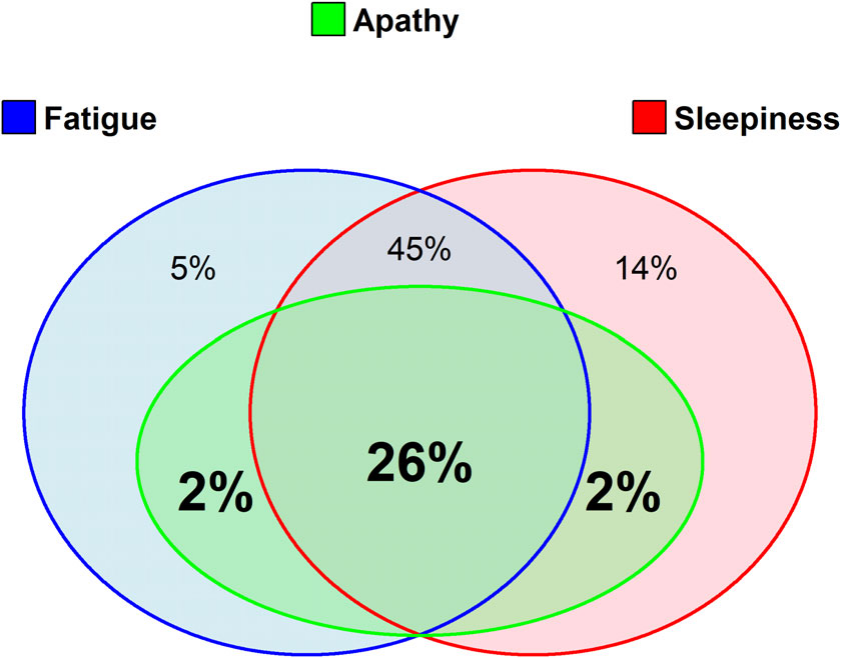
High co‐occurrence of apathy, fatigue, and daytime sleepiness in Parkinson's disease. This Venn diagram illustrates the overlap of patient‐reported apathy (green), fatigue (blue), and daytime sleepiness (red) in a cohort of 199 participants. Among the 59 participants who reported apathy, 53 (89.8%) also reported experiencing both fatigue and sleepiness.

Among 199 participants who completed MDS‐UPDRS testing, 59 (29.6%) reported apathy, which almost exclusively co‐occurred with fatigue or sleepiness (89.8%; Fig. 1). The range and frequencies of scores are depicted in Figure S1.

Subsequent analyses included the 169 participants with complete cognitive (SDMT, Stroop) and psychiatric (HAM‐A, HAM‐D) data (Appendix S1). Of these, 30.8% (52 participants) had apathy. Clinically significant differences were observed between off‐medication and on‐medication states in 14.2% (HAM‐D), 24.9% (HAM‐A), 49.1% (SDMT), and 33.1% (Stroop).

### Network Analysis

**Figure 2:**
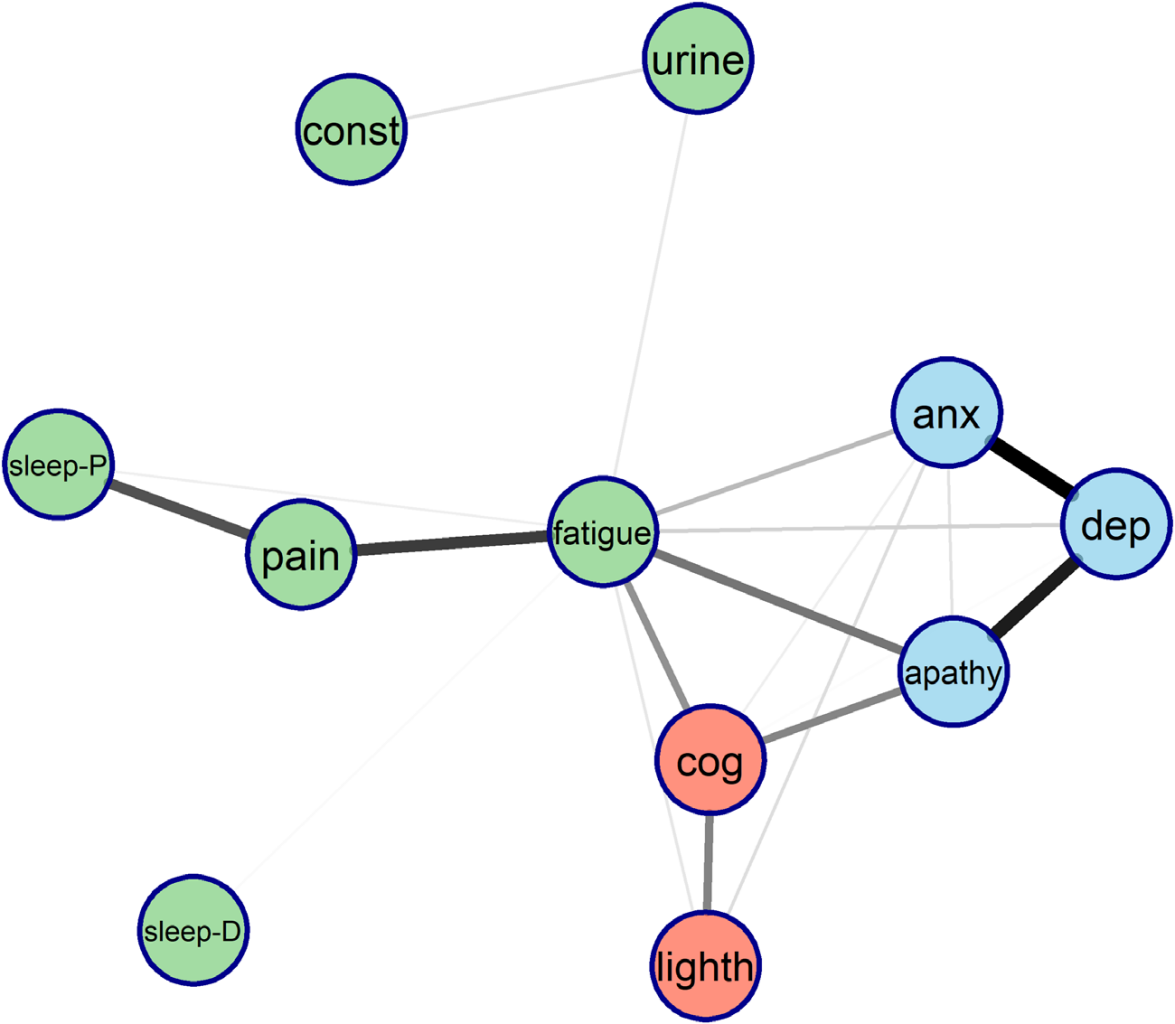
Network of non‐motor symptoms. Nodes represent individual symptoms or scale scores. Edges represent regularized partial correlations between nodes; thicker, darker edges indicate stronger connections. Node colors denote community assignments as identified by the Leiden algorithm. Node labels are abbreviated for clarity. MDS‐UPDRS Part 1 items include: cog (cognitive impairment, 1.1), dep (depressed mood, 1.3), anx (anxious mood, 1.4), apathy (apathy, 1.5), sleep‐P (sleep problems, 1.7), sleep‐D (daytime sleepiness, 1.8), pain (pain and other sensations, 1.9), urine (urinary problems, 1.10), const (constipation, 1.11), and lighth (light headedness on standing, 1.12).

Graph networks were generated (Fig. 2). Stability bootstrap analysis yielded a CS‐coefficient of 0.281 for edge weights and strength centrality, indicating modest stability and exploratory structural inferences.

**Figure 3:**
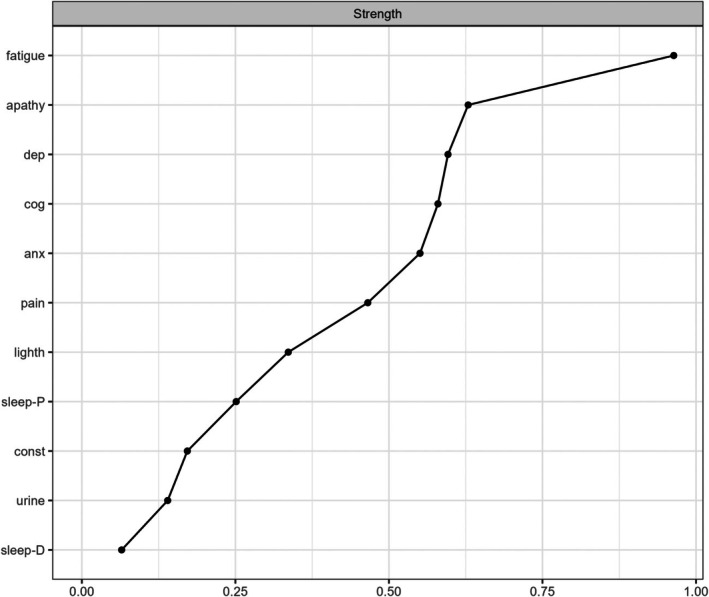
Strength centrality of nodes in the final network. The plot displays the standardized strength value for each symptom node in the network. Nodes are ranked from least central (bottom) to most central (top). Higher values on the x‐axis indicate greater overall connectivity within the network. Node labels are abbreviated as in Figure 5.

In the resulting network graph, the central symptoms in the graph were fatigue, apathy, depression, cognition, and anxiety, and the least central was daytime sleepiness (Fig. 3 and Table S1).

### Network Communities

Community detection analysis partitioned the network into three distinct clusters, including (1) apathy, anxiety, depression; (2) cognition and lightheadedness; (3) fatigue, urinary problems, constipation, pain, sleep problems, and daytime sleepiness. Communities are visualized by colors (Fig. 2).

### Apathy and Medication‐Related Neuropsychiatric Change

Of the 52 participants with apathy, 44 (84.62%) had clinically meaningful medication‐related changes in psychiatric or cognitive symptoms. 24 (46.2%) had change in anxiety, 8 (15.4%) in depression, 27 (51.9%) in SDMT, and 18 (34.6%) in Stroop. Medication‐related anxiety change was significantly more frequent in those with apathy (*χ* ^2^ = 16.64, *P* < 0.001).

### Associations

**Figure 4:**
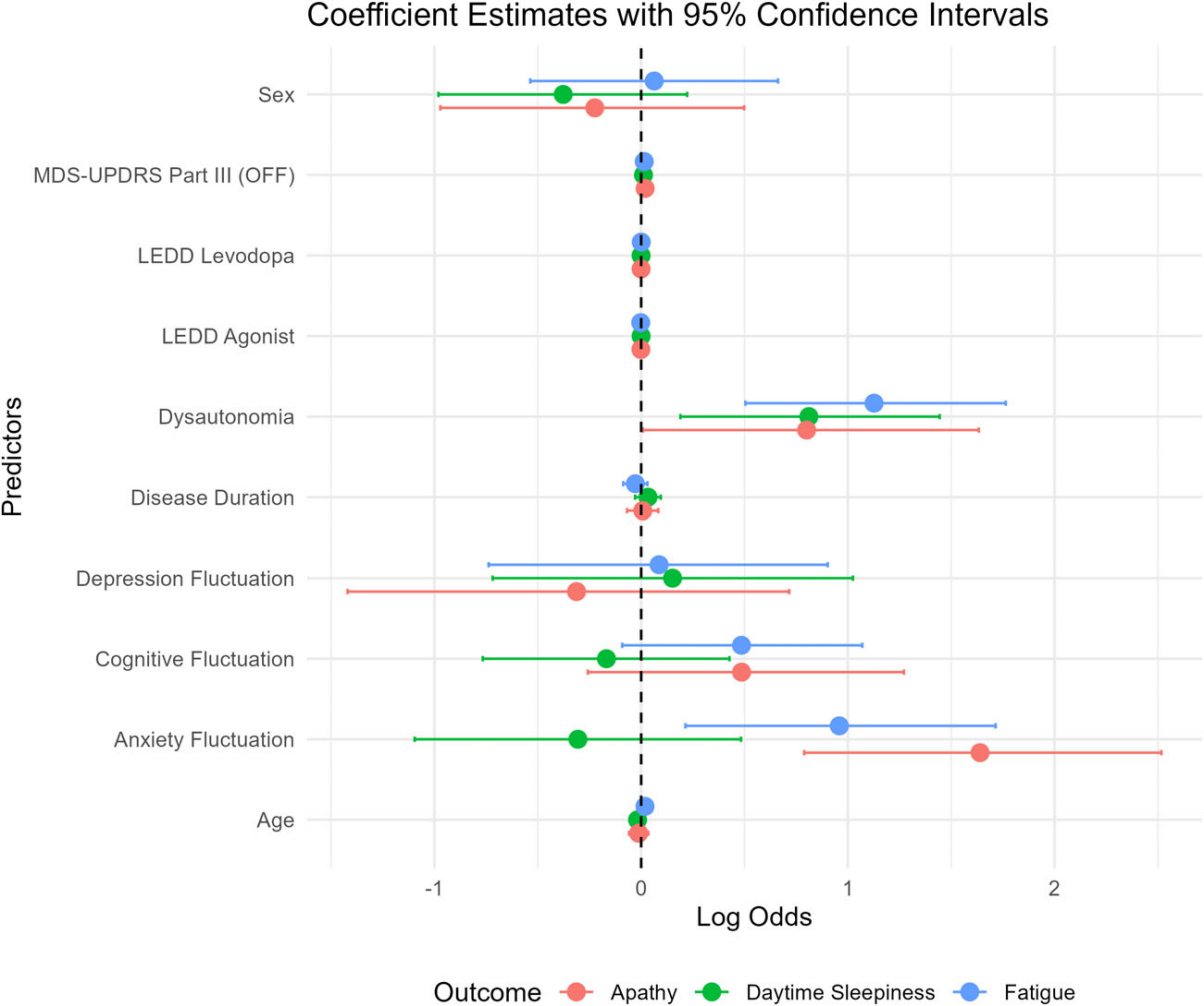
Forest plot showing coefficient estimates (log odds) and 95% confidence intervals for predictors of apathy, daytime sleepiness, and fatigue (each modeled on their original 0–4 ordinal scales) from the respective questions on the MDS‐UPDRS Part I. Predictor variables include age, sex, duration of disease, the MDS‐UPDRS Part III motor score off‐medication, the binarized presence or absence of dysautonomia, as well as binarized presence of clinically meaningful change in anxiety, depression, or cognition between off‐medication and on‐medication states.

We performed an ordinal logistic regression for each patient‐reported outcome measure of apathy, fatigue, and daytime sleepiness (Table 1, Fig. 4). Apathy was associated with change in anxiety from off to on medication state (*β* = 1.57, 95% CI 0.74–2.42, *P* < 0.001, adjusted *P* = 0.007). Fatigue was independently associated with the presence of dysautonomia (*β* = 0.96, 95% CI 0.33–1.61, *P* = 0.004, adjusted *P* = 0.009). Multiple significant associations were observed prior to correction, including daytime sleepiness with dysautonomia (*β* = 0.70, 95% CI 0.06–1.35, *P* = 0.034), and fatigue with both change in cognition (*β* = 0.66, 95% CI 0.06–1.28, *P* = 0.034) and change in anxiety (*β* = 1.69, 95% CI 0.51–3.21, *P* = 0.01); however, these exploratory associations did not survive FDR adjustment.

**TABLE 1:** Regression models of daytime sleepiness, apathy, and fatigue

	Daytime sleepiness		Apathy		Fatigue	
	Beta	*P*‐Value	Beta	*P*‐Value	Beta	*P*‐Value
Change in anxiety	−0.390	0.342	1.569	<0.001*	1.69	0.005
Change in depression	0.165	0.717	−0.592	0.301	0.187	0.658
Change in cognition	−0.123	0.696	0.244	0.536	0.662	0.034
Dysautonomia	0.702	0.034	0.731	0.081	0.963	0.004*
Age	−0.003	0.893	−0.032	0.208	0.034	0.093
Sex	−0.421	0.179	−0.248	0.511	−0.040	0.898
Duration of disease	0.018	0.582	0.000	0.992	−0.030	0.328
MDS‐UPDRS Part III (OFF‐med)	0.013	0.326	0.013	0.359	0.020	0.100

*Note*: The table displays beta coefficients (*β*) and *P*‐values from three separate ordinal logistic regression models. Each column represents a model for one of the three non‐motor outcomes (Daytime Sleepiness, Apathy, or Fatigue). The predictor variables used in each model are listed in the rows. *P* < 0.05 was considered statistically significant. Asterisks (*) denote associations that remained statistically significant after Benjamini‐Hochberg False Discovery Rate (FDR) correction for multiple comparisons.

**Figure 5:**
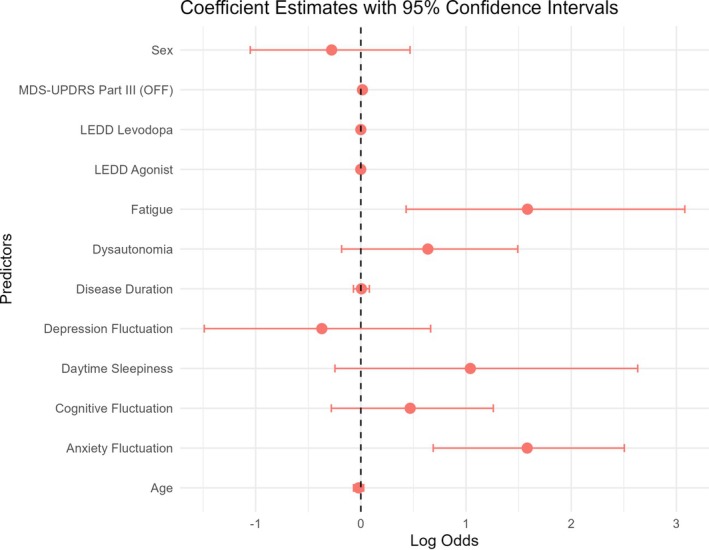
Sensitivity analysis for predictors of apathy, including fatigue and sleepiness. This forest plot displays the coefficient estimates (log odds) and 95% confidence intervals from an ordinal logistic regression model with patient‐reported apathy (modeled on its original 0–4 scale) as the outcome. This sensitivity analysis includes the binarized presence of fatigue and daytime sleepiness as predictor variables to test whether the association between apathy and dopamine medication‐related change in anxiety persists after adjusting for these highly co‐occurring symptoms. A confidence interval that does not cross the zero line (dashed) indicates a statistically significant association (p < 0.05).

In a sensitivity analysis model predicting apathy while adjusting for daytime sleepiness and fatigue (Fig. 5), significant independent associations were observed for change in anxiety (*β* = 1.50, 95% CI 0.62–2.40, *P* = 0.001) and fatigue (*β* = 3.01, 95% CI 1.66–4.48, *P* < 0.001), indicating an independent association between apathy and change in anxiety even when accounting for the impact of daytime sleepiness and fatigue. Furthermore, in a sensitivity analysis using a stricter threshold for clinical apathy (score ≥2), the independent association between apathy and medication‐related change in anxiety remained significant (OR 1.68, 95% CI 0.65–2.75, *P* = 0.002). Multicollinearity among predictor values was assessed across all models; variance inflation factor (VIF) values remained below 1.5, indicating no significant multicollinearity.

### SSRI Use

Of 169 participants, 37 reported SSRI use (Appendix S3). When added as a covariate to the primary apathy model, SSRI use showed a trend toward significance (*β* = 0.75, 95% CI −0.001–1.49, *P* = 0.05), while the association between change in anxiety and apathy remained robust (*β* = 1.57, 95% CI 0.73–2.44, *P* = 0.0003). In the fully adjusted model additionally controlling for daytime sleepiness and fatigue, SSRI use was not significant (*P* = 0.10), and all primary associations were maintained, including change in anxiety (*β* = 1.48, 95% CI 0.59–2.39, *P* = 0.001), and fatigue (*β* = 1.68, 95% CI 0.50–3.21, *P* < 0.001).

### LEDD

In sensitivity analyses adjusting for total and dopamine agonist associated levodopa equivalent daily dose (LEDD), the association between apathy and medication‐related anxiety change remained robust (*β* = 1.64, 95% CI 0.79–2.52, *P* < 0.001). Fatigue (*β* = 1.12, 95% CI 0.50–1.76, *P* < 0.001) and daytime sleepiness (*β* = 0.81, 95% CI 0.19–1.44, *P* = 0.01) retained their associations with dysautonomia, though the relationship between fatigue and cognitive change was no longer significant (*P* = 0.10). Fatigue showed a weak negative association with dopamine agonist LEDD (*β* = −0.002, 95% CI −0.004 to −0.0001, *P* = 0.046) that did not survive FDR correction (*P* = 0.23).

## Discussion

We deconstructed the clinically challenging triad of apathy, fatigue, and daytime sleepiness in PD, a symptom cluster in our data with 90% of apathetic individuals reporting both companion symptoms. In our cohort, 29.6% met criteria for apathy, modestly lower than two published estimates of 39.8%^4^ and 38%.^56^ This possibly reflects the apathy metric used^57^ and our cohort's comparatively milder disease severity due to the exclusion of dementia and the necessity of patients tolerating repeat testing for study inclusion.

Exploratory network analyses suggest partially overlapping yet dissociable symptom clusters, with fatigue reflecting a broader somatic profile linked to dysautonomia and pain, with daytime sleepiness occupying a peripheral position, and where apathy aligns more closely with affective symptoms. In this context, apathy shows an association with dopamine‐medication‐related change in anxiety that remains independent of fatigue and sleepiness, indicating a specific affective‐motivational linkage and suggesting dopaminergic instability in mesocorticolimbic circuits.^17^, ^18^, ^19^

Medication‐related change in this study denotes a single within‐visit off to on‐medication difference. Apathy and fatigue may be less responsive to overnight washout or single‐dose levodopa than anxiety or motor signs, so repeat sampling will be needed to determine whether stabilizing dopaminergic tone reduces their day‐to‐day variability. We hypothesize that in susceptible individuals, the pulsatile nature of oral levodopa therapy leads to lability in these shared circuits, manifesting concurrently as changes in anxiety^39^ and goal‐directed behavior.

Apathy's association with medication‐related anxiety aligns with increased apathy in off‐‐states^15^ and orbito‐medial prefrontal‐basal ganglia models.^25^ Importantly, this association persisted independent of fatigue and after adjusting for total and agonist‐specific LEDD, indicating that the relationship is driven by dynamic levodopa responsiveness rather than a static daily dose. Fatigue also associated with change in anxiety suggesting minor but distinct dopaminergic sensitivity compared to the more robust levodopa‐responsiveness of the apathy‐anxiety cluster. Clinically, continuous dopaminergic stimulation may help stabilize these circuits, even if anxiety benefits diverge from motor response.^58^

Fatigue emerged as a systemic symptom most strongly linked to pain, apathy, perception of cognitive impairment, and dysautonomia.^32^ This implicates brainstem noradrenergic contributions^34^ and the cumulative burden of non‐motor symptoms as drivers of fatigue.^7^, ^34^ Notably, sensitivity analysis revealed a weak negative association between fatigue and dopamine agonist LEDD (*β* = −0.002, *P* = 0.046), suggesting that while fatigue is primarily driven by non‐dopaminergic somatic burden, it may maintain a minor sensitivity to specific receptor stimulation (agonists), distinct from the acute response to levodopa. By contrast, apathy showed a tighter affective linkage with associations to anxiety and depression, supporting a cortico‐limbic‐striatal mechanism distinct from fatigue's somatic profile. Daytime sleepiness occupied a peripheral position with weak links to other symptoms and no independent association with apathy after adjusting for fatigue, supporting a separable clinical entity, perhaps driven by primary sleep–wake mechanisms rather than mood or motivational state.

Our findings underscore the necessity of viewing apathy in PD as a complex syndrome arising from multiple neurochemical systems and clinically overlapping with other non‐motor symptoms.

Selective serotonin reuptake inhibitors (SSRIs), commonly prescribed for anxiety and depression in PD, have themselves been implicated in the development or worsening of apathy.^24^, ^59^ A bidirectional relationship may also be considered with apathy, misdiagnosed as depression, being treated with SSRIs. In our sensitivity analysis, the addition of SSRI use as a covariate in models predicting apathy demonstrated a trend toward significance, though this association was not sustained in the sensitivity analysis with additional adjustment for fatigue and daytime sleepiness. Notably, in these fully adjusted models, fatigue, sleepiness, and change in anxiety all remained significant predictors of apathy. These findings are in line with prior research reporting a clinical relationship between SSRI use and apathy,^24^, ^59^ but further support the concept that fatigue, daytime sleepiness, and change in anxiety each have independent associations with apathy in PD.

From an exploratory systems‐level view, the strongest edges in this specific cohort's network linked depression with anxiety and sleep problems with depression, mirroring well‐described clinical co‐occurrence.^60^, ^61^ The third strongest edge connected apathy and fatigue, suggesting these symptoms may be mutually reinforcing. Network analysis identified a stronger direct edge between apathy and depression than between apathy and anxiety, consistent with their established symptomatic overlap. However, our regression models captured a distinct relationship: apathy's association with medication‐related change in anxiety, indicating that static co‐occurrence tracks with depression, but the dynamic, dopamine‐dependent modulation of apathy is coupled with anxiety fluctuations. Fatigue's broad coupling with multiple non‐motor symptoms including pain, a previously reported association in PD,^32^ suggests that it may function as a pathogenic amplifier that disproportionately shapes overall network architecture. If confirmed in longitudinal studies, therapeutic strategies targeting fatigue and its somatic correlates may yield benefits extending beyond the targeted symptom.

The primary connections to the apathy node in our network empirically map onto its classic multidimensional constructs:^25^, ^62^ an (1) emotional‐affective component tied to depression and anxiety in dopamine‐sensitive limbic‐medial prefrontal circuits,^25^, ^38^, ^39^, ^63^ a (2) behavioral/auto‐activation component intertwined with fatigue‐related initiation costs, and a (3) cognitive component linked to subjective cognitive impairment that may reflect executive network dysfunction.^64^, ^65^, ^66^ While requiring validation in larger samples, these domain‐specific associations raise the possibility of targeted therapeutic approaches: serotonergic or noradrenergic strategies for affective presentations, behavioral activation therapy for fatigue‐driven failure of initiation, and cholinergic augmentation for executive‐motivational deficits.^26^, ^64^, ^65^, ^66^, ^67^ Alternative explanations remain possible, including that anxiety‐related avoidant behavior may be endorsed as apathy on self‐report, underscoring the need for formal scale‐based differentiation.

Despite daytime sleepiness's peripheral network position, the strong clinical overlap remains, with 90% of our apathetic participants also reporting fatigue and sleepiness. The high co‐occurrence suggests that therapeutically targeting fatigue or sleepiness may yield downstream benefits for apathy through shared neurochemical pathways.

### Limitations

This study used single MDS‐UPDRS line items to assess apathy, fatigue, and sleepiness. Reliance on a single item (especially of mild severity ratings) risks capturing non‐specific symptoms and lacks the specificity of comprehensive scales. However, a sensitivity analysis with a stricter threshold for apathy (≥2) confirmed the independent association between change in anxiety and apathy. Furthermore, we observed lack of multicollinearity between the three symptoms, and there is also literature demonstrating strong correlation of the apathy item with the Lille Apathy Rating Scale (rho = 0.516, *P* < 0.001).^68^ We assessed the stability of our network model using a case‐dropping bootstrap approach and made informed adjustments to enhance stability; however, the centrality stability coefficient (CS = 0.281) indicates modest stability, and centrality estimates should therefore be interpreted with caution.

Additionally, apathy, fatigue, and sleepiness were assessed over the preceding week without medication‐state specification, preventing direct measurement of their fluctuations. Nevertheless, our use of acute pharmacodynamic responses in anxiety and cognition as probes of dopamine‐sensitive mechanisms revealed that apathy, unlike fatigue and sleepiness, tracks with these acute changes, supporting a dopamine‐sensitive component. The HAM‐A and HAM‐D were designed to assess symptoms over days to weeks. It is possible that these instruments, particularly the HAM‐D and to a lesser extent the HAM‐A, were not sufficiently sensitive to capture acute changes with dopaminergic replacement, thereby underestimating the effect. To minimize this, we administered these measures with a pre‐planned modification that queried the patient's “current state” when possible. Regardless, items inherently tied to longer timeframes (eg, insomnia, guilt, weight loss) are not amenable to capturing rapid state changes, thereby perhaps underestimating the magnitude of dopamine‐related affective fluctuations.

Generalizability may be limited by several features of the parent study design. This dataset was collected to evaluate acute dopaminergic responses, so medication‐naïve patients were excluded. Individuals unable to tolerate repeat OFF/ON testing were also excluded, which may limit applicability to more advanced PD populations. However, no global cognitive screening cutoff was applied, and the only cognitive exclusion was a clinical diagnosis of dementia, preserving a relatively broad and representative sample of PD patients across disease stages. Finally, data on primary sleep disorders such as sleep apnea and restless legs syndrome were unavailable, limiting control for contributors to daytime sleepiness. Future work should employ longitudinal, multi‐domain scales to refine these relationships and inform therapeutic strategies that remain limited in efficacy.^26^

## Summary

The complex interplay and high co‐occurrence of apathy, fatigue, and daytime sleepiness in PD complicate clinical assessment. While exploratory, the data‐driven network perspective we present suggests fatigue and apathy may act as central and interlinked features within this cohort, whereas daytime sleepiness remains peripheral. Apathy shows a unique association with clinically significant, medication‐related anxiety change, independent of fatigue and sleepiness, implicating dopamine‐sensitive cortico‐limbic motivation circuits. By contrast, fatigue and sleepiness align with dysautonomia and broader somatic burden, highlighting modifiable targets such as autonomic symptoms, primary sleep disorders, and pain. The results highlight apathy's strong centrality among PD non‐motor symptoms, its shared yet distinct relations to fatigue and sleepiness, and a specific association with dopamine medication related anxiety change.

## Author Roles

(1) Research Project: A. Conception, B. Organization, C. Execution; (2) Statistical Analysis: A. Design, B. Execution, C. Review and Critique; (3) Manuscript Preparation: A. Writing of the First Draft, B. Review and Critique

J.S.: 1A, 1B, 1C, 2A, 2B, 2C, 3A, 3B.

A.M.: 1B, 2A, 2C, 3B.

C.M.: 1B, 2A, 2C, 3B.

J.M.: 2A, 2C, 3B.

D.B.: 2A, 2C, 3B.

K.M.: 1A, 1B, 1C, 2A, 2C, 3B.

G.P.: 1A, 1B, 1C, 2A, 2C, 3B.

## Disclosures

**Ethical Compliance Statement:** The Johns Hopkins University Institutional Review Board approved the study protocol for data collection. All participants provided written informed consent before participation. We confirm that we have read the Journal's position on issues involved in ethical publication and affirm that this work is consistent with those guidelines.

**Funding sources and conflicts of interest:** G.M. Pontone and the study described in the manuscript were funded by the NIH/NIA as part of a K23 award (K23AG044441). The authors declare that there are no conflicts of interest relevant to this work.

**Financial Disclosures for the Previous 12 Months:** A.M. has received funding from Parkinson's foundation, Sunflower Parkinson's disease foundation, and Dystonia Medical Research Foundation, and he has served on the advisor board of Adaptive Biosciences through the Parkinson's Study Group. He serves as a consultant for Vima Therapeutics Inc. He serves as an associate editor for *Movement Disorders Clinical Practice*. C.B.M. has received funding from the National Institutes of Health (K23AG088248, KL2TR003099, L30AG083912). K.A.M. has received funding from Parkinson's Foundation, The Michael J. Fox Foundation, and NINDS/NIH (R21NS128391‐01A1). He receives clinical trial research support from UCB Pharma. G.P. was funded by 5K23AG044441 during the completion of this work and is now funded by 1R01MH123552; he has also carried out consulting with Acadia Pharmaceuticals Inc and GE Healthcare. The remaining authors report no conflict of interest.

## Supporting information


**Appendix S1.** Missing Data. Description of data handling for participants with incomplete testing.
**Appendix S2.** Cognitive Testing. Details on the administration and scoring of the SDMT and Stroop tests.
**Appendix S3.** SSRI Analysis. Methodology for the sensitivity analysis involving Selective Serotonin Reuptake Inhibitor (SSRI) use.
**Figure S1.** Symptom Frequency. The frequency of apathy (green), excessive daytime sleepiness (red), and fatigue (blue) across scores 0–4 derived from the MDS‐UPDRS Part I. All participants (n = 200) are included in this analysis
**Table S1.** Network model edge weights. The table displays all edges present in the final network model, sorted by weight in descending order. Weights represent the regularized partial correlation between two nodes after accounting for all other nodes in the network. Node labels correspond to items from the MDS‐UPDRS Part I and are as follows: Cognition (1.1); Depression (1.3); Anxiety (1.4); Apathy (1.5); Sleep Problems (1.7); Daytime Sleepiness (1.8); Pain (1.9); Urinary Problems (1.10); Constipation (1.11); Lightheadedness (1.12); and Fatigue (1.13)

## Data Availability

The data that support the findings of this study are available from the corresponding author upon reasonable request.
