# Supplementary material for: Apathy in Parkinson's Disease: Distinguishing Overlapping Symptoms Via Network Analysis
Source: Mov Disord Clin Pract. 2026 Jun 2:10.1002/mdc3.70698. Online ahead of print. doi: 10.1002/mdc3.70698 (PMC13339150; doi:10.1002/mdc3.70698)
Supplement: Supplementary file 1 — Appendix S1. Missing Data. Description of data handling for participants with incomplete testing. Appendix S2. Cognitive Testing. Details on the administration and scoring of the SDMT and Stroop tests. Appendix S3. SSRI Analysis. Methodology for the sensitivity analysis involving Selective Serotonin Reuptake Inhibitor (SSRI) use. Figure S1. Symptom Frequency. The frequency of apathy (green), excessive daytime sleepiness (red), and fatigue (blue) across scores 0–4 derived from the MDS‐UPDRS Part I. All participants (n = 200) are included in this analysis Table S1. Network model edge weights. The table displays all edges present in the final network model, sorted by weight in descending order. Weights represent the regularized partial correlation between two nodes after accounting for all other nodes in the network. Node labels correspond to items from the MDS‐UPDRS Part I and are as follows: Cognition (1.1); Depression (1.3); Anxiety (1.4); Apathy (1.5); Sleep Problems (1.7); Daytime Sleepiness (1.8); Pain (1.9); Urinary Problems (1.10); Constipation (1.11); Lightheadedness (1.12); and Fatigue (1.13) [file MDC3-9999-0-s001.docx]

**SUPPLEMENTARY DATA:**

**Supplementary Appendix 1: Missing Data**:

This study uses a dataset that was originally collected to study off and on-medication changes in anxiety which has been previously published.^68^ In total, 200 subjects were initially included in the study.

199 of the 200 subjects completed MDS-UPDRS testing. One subject skipped the MDS-UDPRS question 1.8 on daytime sleepiness. Therefore, this participant was omitted from our analysis.

172 participants completed Stroop testing and 181 completed SDMT. In total, there were 171 participants (in the full 200 participant dataset) who completed both Stroop and SDMT testing. As the dataset was originally created to obtain psychiatric measures ^46^, The cognitive tests were added after initial data collection started on the project, leading to 18 missed participants for both tests. One additional participant could not complete either test due to fatigue. Additionally, nine Stroop values were not completed. For eight of these nine additional missing Stroop cases, the reason was color blindness, and for the remaining one of the nine, they could not tolerate repeat Stroop testing due to fatigue.

One patient did not complete Hamilton depression scale (HAM-D) testing due to omission of one question in the scale.

In total, there were 169 patients who had complete cognitive (Stroop, SDMT), psychiatric (Hamilton depression and anxiety) and MDS-UPDRS data for inclusion in our regression models in this study.

**Supplementary Appendix 2: Cognitive Testing**:

The SDMT was administered orally to prevent confounding due to motor slowing. The timed Stroop measured the number of correct responses completed in 45 seconds during three trials: color naming, word naming, and color-word interference naming. Both tests assess multiple cognitive domains, including processing speed, working memory, attention, and executive function.

There are several missing cognitive test scores in the dataset. This is because some subjects did not tolerate repeat cognitive testing, and some entered the study prior to the addition of the cognitive testing to the protocol (refer to above section on missing data in this supplement).

**Supplementary Appendix 3: SSRI Analysis**:

Methods: The use of SSRI medication was included in a sensitivity analysis of our study. We screened the following SSRI medications: citalopram, escitalopram, fluoxetine, fluvoxamine, paroxetine, sertraline, and vilazodone.

Of the 169 participants in our study’s regression model, 37 patients reported SSRI use, including: escitalopram (13), sertraline (11), citalopram (5), fluoxetine (5), paroxetine (2), and vilazodone (1).

**Supplementary Figures:**


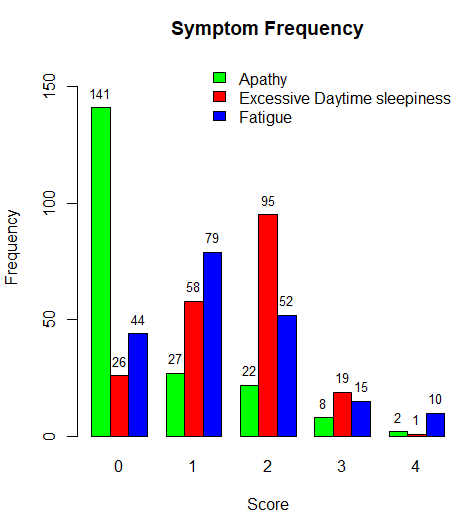


**Supplemental Figure S1**: The frequency of apathy (green), excessive daytime sleepiness (red), and fatigue (blue) across scores 0-4 derived from the MDS-UPDRS Part I. All participants (n=200) are included in this analysis.

**Supplementary Table S1:** **Strength of edges in the network model of PD non-motor symptoms**

| **Node 1** | **Node 2** | **Weight** |
| --- | --- | --- |
| Depression | Anxiety | 0.241 |
| Depression | Apathy | 0.220 |
| Pain | Fatigue | 0.196 |
| Sleep Problems | Pain | 0.180 |
| Apathy | Fatigue | 0.154 |
| Cognition | Lightheadedness | 0.143 |
| Cognition | Apathy | 0.142 |
| Cognition | Fatigue | 0.132 |
| Anxiety | Fatigue | 0.101 |
| Depression | Fatigue | 0.083 |
| Anxiety | Lightheadedness | 0.075 |
| Urinary Problems | Constipation | 0.073 |
| Anxiety | Apathy | 0.071 |
| Lightheadedness | Fatigue | 0.068 |
| Urinary Problems | Fatigue | 0.066 |
| Cognition | Anxiety | 0.062 |
| Sleep Problems | Fatigue | 0.062 |
| Daytime Sleepiness | Fatigue | 0.054 |
| Cognition | Depression | 0.052 |
| Pain | Lightheadedness | 0.050 |
| Constipation | Fatigue | 0.048 |
| Cognition | Constipation | 0.041 |
| Apathy | Pain | 0.032 |
| Apathy | Daytime Sleepiness | 0.011 |
| Sleep Problems | Constipation | 0.010 |
| Cognition | Pain | 0.008 |

**Supplementary Table Notes:** The table displays all edges present in the final network model, sorted by weight in descending order. Weights represent the regularized partial correlation between two nodes after accounting for all other nodes in the network. Node labels correspond to items from the MDS-UPDRS Part I and are as follows: Cognition (1.1); Depression (1.3); Anxiety (1.4); Apathy (1.5); Sleep Problems (1.7); Daytime Sleepiness (1.8); Pain (1.9); Urinary Problems (1.10); Constipation (1.11); Lightheadedness (1.12); and Fatigue (1.13).
